# Identification of novel inhibitor targeting Fyn kinase using molecular docking analysis

**DOI:** 10.6026/97320630015419

**Published:** 2019-06-15

**Authors:** Dharani Nedunchezhian, Kulanthaivel Langeswaran, Sundar Santhoshkumar

**Affiliations:** 1 Department of Biotechnology and Bioinformatics,Bishop Heber College,Bharathidasan University,Tiruchirapalli,Tamil Nadu,India; 2 Cancer Genetics and Molecular Biology Laboratory, Department of Bioinformatics, Science Campus, Alagappa University, Karaikudi, Tamil Nadu, India; 3 Department of Computer Science, Science Campus, Alagappa University, Karaikudi, Tamil Nadu, India

**Keywords:** Tyrosine fyn kinase, ligand binding, dynamic simulation

## Abstract

Identification of tyrosine Fyn kinase inhibitor is recognized as an effective and feasible therapeutic measure in reducing consequences of memory loss disorder Alzheimer's. The present investigation has been attempted with an objective to find out a novel potent inhibitor with similar homological structure to Fyn kinase using structure based in silico screening measure. Such derived structure was compared with natural data base pool and were systematically analyzed. Ligand based interaction was also tested and evaluated. We applied a molecular dynamic simulation technique to validate the stability of the identified complexes and to understand the ligand binding mechanism. Results provide information on the characteristics of novel and potent inhibitor for tyrosinase Fyn kinase protein so as to develop an innovative strategy to treat AD.

## Background

Tyrosine Fyn Kinase is a determined protein factor involved in the activity of cell signaling pathway. Unusual occurrence in the rate of activity of Fyn Kinase in signaling, render toxic substances, resulting chronic disorder such as Alzheimer's 1. This played a crucial role in phosphorylation and anti-phosphorylation process and confers its intra molecular affinity 2, Cell adhesion, synaptic activity and signaling 3. Several earlier reports were demonstrated and evident that Tyrosine Fyn was recognized as driven force causing AD by interacting Amyloid B and Tau, conferring a novel therapeutic strategy to address and minimize the consequences of AD. Regulation of Fyn activity was obviously studied in several types of signaling pathways 4. Shirazi et al. 5 have reported that Fyn Protein showed immune reactivity while unusual phosphorylated Tau protein. However, several studies were focused on inter linkage of Tyrosine Fyn Kinase protein with AD. Participation of Fyn Kinase Protein in inducing and act as causative agent for chronic disorder. Therefore, it received more attention on its ability in signaling pathways. Advancement of docking based virtual screening technique in accordance with affinity of protein and ligand conferring an ideal way for screening the lead novel compounds in the field of molecular docking 6. Identification of macromolecules and validity was the remarkable factor in targeting the drugs, however, it is still unclear and debatable 7. Therefore, in the present investigation, a trial has been taken up (i) to predict the novel potent inhibitor targeting Fyn Kinase and (ii) to identify the ligand by comparing natural database and (iii) to explore protein ligand complex stability and its behavior. 

## Methodology

Using Software packages such as CPU platforms performed aforementioned tasks. Stipulations were CPU-Supermicro 32Gb Nvidia Quadro K620 with 2GB included, LLC, 2019 Desmond. Fyn protein complex code (PDB ID-2DQ7) with a resolution of 2.8 Armstrong was used for virtual screening and molecular docking. They contain heavy atoms, covering its cofactors. However, to avoid left out information on connectivity, protein structure is imported from PDB into Maestro in the protein preparation wizard with hydrogen polar. Optimization of Hydrogen bond was employed and rely on clusters of hydrogen-bonded species were selectively optimized. Minimizing the structure has been performed to refine the structure, with a restrained minimization. The RMSD value of the atom was minimized. The partial atomic charges were also computed and analyzed. 

The generated structure of a prepared protein showed in an order such as receptor, site, constraints and rotatable groups. Receptor is a workspace and the ligandwere identified as a molecule. The green color marker should be shown after the ligand molecule is picked in the workspace. Van der Waals (vdW) radius scaling of 14 for closely contacts with the ligand and the receptor (a protein molecule) is used. Scaling factor default value is 0.30 and the partial charge cut off default value is 0.25. Scaling of van der Waals radii was performed on the non-polar atoms. Scale factor default value is 1.0 as the contact by small gaps in the solvent-exposed regions and also determines where in the scoring grids are located. Energetic program was used to analyses spectrum to predict the modes of the protein ligand complexes. Workflow filtering method was used. It was also evaluated the acceptability of analogues based on Lipinski's rule of 5 which was essential to ensure the drug-like properties of the molecules were assessed. GSAP complexed with compounds from Zinc-NPD, NPB, Nci-NPD database was employed by using the Desmond modular. In this present study, totally three from Zinc-NPD, NPB, Nci-NPD database (144027, 2455, 9219) were chosen, theses three are the top leads from each database identified based on the docking score. All these three complexes were simulated for 10 analyzed deviations (RMSF) and considered as backbone of the protein. The RMSF was assessed to estimate fluctuation. 

## Result and Discussion:

**Figure 1:**
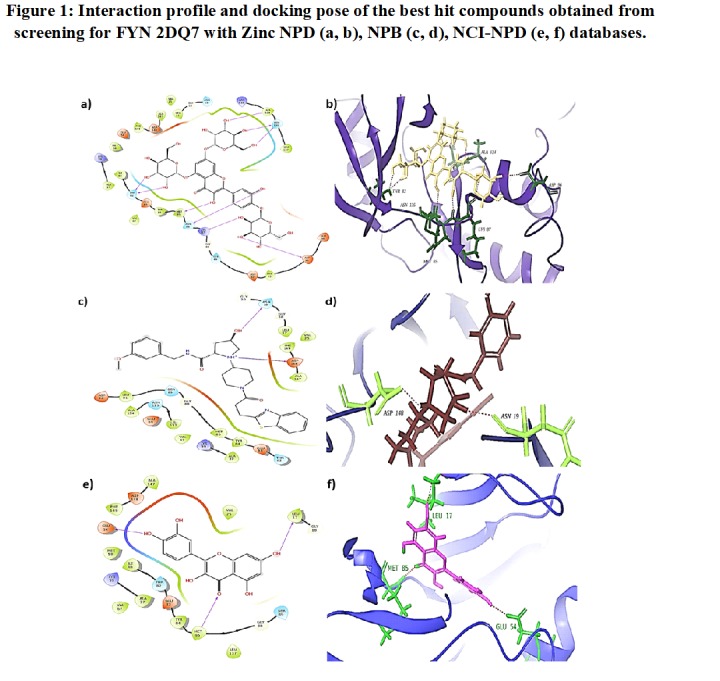
Interaction profile and docking pose of the best hit compounds obtained from screening for FYN 2DQ7 with Zinc NPD(a, b), NPB(c, d), NCI-NPD(e, f) databases

In the present study high throughput virtual screening (HTPS) method was used to identify potential molecule inhibitors using compounds obtained from pharmacophore against active FYN kinase protein of the ligands were further docked flexibly by XP which generates compounds were tabulated in Table 1 and shown in Figure 1. Drug likeness was tested and followed by Quikprop method of Schrodinger ADME properties of identified inhibitors were recorded systematically and tabulated (Table 1) based on Lipiskis rule of 5 8. Results of the docking indicated that efficient inhibitors identified among three database, chief lead compound was found to be G Plog Po and was promising alternate. Final results of screened compounds were selected data base as an input to find matches to hypothesis to screen the best ligands molecules. In earlier studies, virtual screening molecules against several proteins related to human disorder were reported. Poli et al. 9 have demonstrated that few efficient Fyn inhibitors have identified and proved to be blocked the activities of Fyn kinase.In our study, three different Natural compounds databases were chosen namely ZINC NPD (3,76,884 Ligand molecules), NPB (20,783 Ligand molecules), NCI(56,000 Ligand molecules) were taken for screening process. Based on the docking score top three compounds were selected from each database. 

### Analysis on ADME of inhibitors:

**Figure 2:**
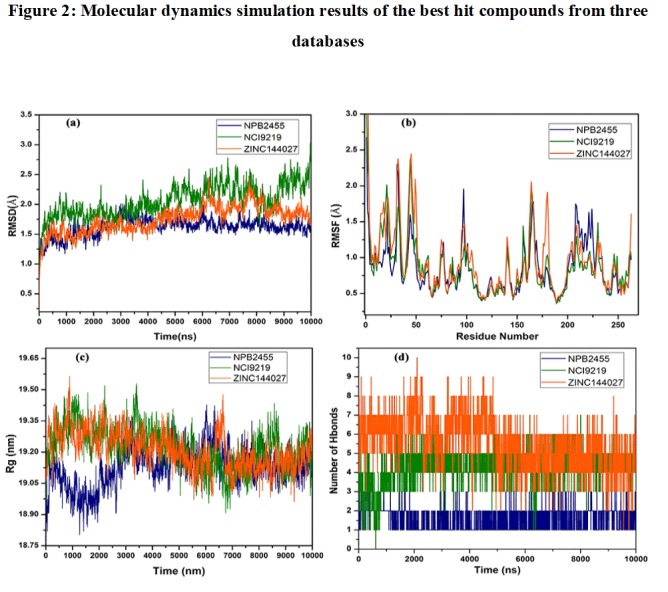
Molecular dynamics simulation results of the best hit compounds from three databases

ADME properties evaluation of identified chief compound, A Plogpo scored highest range (Table 2). It was observed that identified compounds with a group of hydrogen bonding donor and acceptor pair as found This present study results were matching the findings of Zhu 10. The biological membrane access ranges were found to be range from 293.11 to 2288.96 and the range of QPPMDCK was 96.8 to 100 11. Thomas et al. 12 have established that improvement in ADME properties of identified compounds are attributed and translate to the pharmacokinetic properties. Findings were correlated with earlier study results.In the present investigation two ligands from the ZINC-NPD database (95914724), NPB database (2455) and NCI-NPD database (6152) were chosen. 95914724, 2455, 6152 were the top lead identified based on docking score. All two complexes were calculated and analyzed RMED value in order to evaluate deviation in the structure during simulation (RMSD). The stability of protein-ligand (2DQ7-144027, 2DQ7- 2455, 2DQ7-9219) complexes is shown. Figure 2 exhibited that the deviations and fluctuations were not observed much and hence the results were agreeable. These results inferred that the compounds have slight fluctuations, which are observed, only in the loop region of the target protein. Zinc complexes in lead molecules may contribute and involved in coordination if neurons characterized by fast ligand exchange 13. In the present study, it was evident that interaction of compound with zinc was attributed to the selected ligand and it was agreement with our findings 14. The results of the RMSD plot reported that high scored lead compound could be satisfactorily explored in the present simulation experiment.

## Conclusion

We are interested identifying the inhibitors targeting the Fyn kinase for the treatment of Alzheimer's disease. It was concluded that Fyn kinase inhibitors are, therefore promising novel molecules and in disrupting a neurodegenerative disorder. Computational studies such as receptor based screening and molecular dynamics simulation have proved and supported our claims to be the useful tool and the best ligand to block the Fyn kinase. This study has confirmed that these compounds can be a potent inhibitor to block the interactions between the targets and inhibit the progression of Alzheimer's disease.

## Conflict of Interest

The authors declared that there are no conflicts of interest.

## Figures and Tables

**Table 1:** Virtual screening of ZINC-NPD, NPB and NCI results

S. No	Natural compounds	I.DS	GE	VDW	COUL	GEM
	ZINC-NPD					
1	144027	-16.572	-68.639	-37.652	-30.987	-121.633
2	120095	-15.884	-82.871	-46.541	-36.33	-113.64
3	128794	-15.848	-90.119	-58.883	-31.236	-130.885
4	2582	-15.812	-79.383	-52.157	-27.227	-105.107
5	118082	-15.799	-82.457	-55.718	-26.739	-99.998
	NPB					
1	2455	-9.946	-63.013	-51.89	-11.123	-92.357
2	7898	-9.838	-64.901	-41.363	-23.538	-81.703
3	2407	-9.595	-58.628	-47.704	-10.923	-81.459
4	4794	-9.096	-42.552	-39.816	-2.737	-65.886
5	2786	-8.961	-53.323	42.002	-11.321	-72.928
	NCI					
1	9129	-10.713	-46.113	-35.774	-10.339	-68.734
2	6152	-10.199	-35.153	-19.122	-16.031	-48.802
3	4036	-10.026	-39.686	-23.145	-16.541	-52.388
4	9037	-9.874	-38.099	-25.992	-12.107	-65.595
5	2608	-9.845	-35.57	-23.836	-11.734	-61.072

**Table 2:** ADME results of the top compounds

S. No	COMPOUND	MW	DONAR HB	ACCEPT HB	% HOA	Q plogPo/W	QplogHERG	QPP Caco	QPPMDCK
1	ZINC_NPD144027	396.463	1	9	88.588	-5.76.	-5.708	0.235	0.059
2	NPB: 2455	240.258	3	2.25	86.594	2.038	-4.643	463.328	215.385
3	NCI: 9129	478.636	1	10.5	85.564	2.919	-5.399	209.104	128.805

## References

[01] Poli G (2013). J Chem Inf Model..

[02] Kramer-Albers EM (2011). Cell Mol Life Sci..

[03] Resh MD (1998). Int J Biochem Cell Biol..

[04] Hu JL (2010). Mol Brain..

[05] Shirazi SK (1993). Neuroreport..

[06] Nguyen TH (2002). J Biol Chem..

[07] Moellering RE (2009). Nature.

[08] Lipinski CA (2001). Adv Drug Deliv Rev..

[09] Goldsmith JF (2002). Biochem Biophys Res Commun..

[10] Zhu L (2018). Mol Divers..

[11] Thomas SM (1997). Annu Rev Cell Dev Biol..

[12] Thomas R (2015). Pediatrics..

[13] Elena Atrian-Blasco (2017). Dalton Trans..

[14] Monika (2013). Bioinformation.

